# Catarrhal Deafness—A More Favorable Prognosis1Read at the thirty-first annual meeting of the Medical Association of Central New York, at Auburn, October 18, 1898.

**Published:** 1899-08

**Authors:** Sargent F. Snow

**Affiliations:** Syracuse, N. Y.; Aurist and laryngologist to the House of the Good Shepherd, Shelter for Unprotected Girls, and the Syracuse Free Dispensary


### CATARRHAL DEAFNESS—A MORE FAVORABLE PROGNOSIS.[1]

By SARGENT F. SNOW, M. D., Syracuse, N. Y.,

Aurist and laryngologist to the House of the Good Shepherd, Shelter for Unprotected Girls, and the Syracuse Free Dispensary.

THE apathy of the medical profession regarding so-called "catarrhal deafness," the lamentable resignation of the afflicted, and the improved facility for combating this disease, impels me to ask your indulgence for a few moments; further apology I feel

1. Read at the thirty-first annual meeting of the Medical Association of Central New York, at Auburn, October 18, 1898.

is unnecessary. Your sympathies have been too much excited by the deaf, to require excuses for a paper written in the hope that a better prognosis may be hastened.

For many years the chronic type of this affection baffled the skill of the foremost otologists. Each apparent success was dimmed by the progressive nature of the disease, and gradually it took a place in the list of nonpreventable and incurable maladies.

I shall not assume that those almost totally deaf can be much improved, but we must admit that recent advances have changed the prognosis in other conditions, why not in many cases of chronic catarrhal deafness?

A study of the anatomy of the parts shows us the intimate relation of the nasal and aural membranes, both by continuity and sympathy. What benefits the nose, is in the right line to benefit the ear in some degree. It matters not whether we have to deal with an atrophy or a sclerosis, our first duty is to relieve the nasal passages of all abnormal conditions.

The aural affections that accompany or follow la grippe, measles, scarlet fever, and the like, we will pass over rapidly. They are essentially catarrhal, and have extended from the active pharyngeal inflammation always present in those diseases. As you well know, they are very aggressive, frequently causing rupture of the drum-head, or what is sometimes more harmful to the subsequent auditory function, a lymphoid deposit within the middle ear. Either of these conditions are amenable to treatment, if inaugurated promptly, the first by antiseptic cleansing and the second by systematic inflation.

The sudden cases of deafness, caused by ordinary acute or subacute catarrhal inflammations can usually be handled by the general practitioner, if a mild alkaline solution, as Seiler's or Dobelle's, be sprayed into the nose twice a day, and the ear inflated three times a week by the Politzer method, but the most careful attention of a trained specialist is required in the commonly termed incurable chronic cases.

This latter class of patients, recognised by a history of deafness, varying from one to twenty years, which may have progressed insidiously, to a degree where the acuometer can just be heard, and at a distance of two feet we have to ask our questions in a loud distinct voice, is the one deserving earnest consideration, and the most patient, skilful treatment.

During the past seven years, it has been my good fortune to be consulted by many patients whose deafness, by age, tests, duration and degree, was surely caused by a chronic catarrhal state, and whose intelligence enabled me to show them the why and wherefores of the persistent line of treatment necessary in such deep seated cases. A number have been under observation five, three and two years, giving a good opportunity for impartial judgment on the methods in vogue at the present day, and I am glad to say that the principle of treatment, as suggested by such advanced authorities as Greuber, Dench and others, that all pathological conditions within the nose and naso-pharynx must be removed, and suitable stimulative absorbing agents introduced through the Eustachian tubes into the middle ear, have proved to be correct.

The idea is rational, and it is my firm belief that a principal reason for many failures is our lack of courage in recommending patients to persistently submit, from year to year if necessary, to atonic and alterative treatment of the membrane. We have been led to expect too much from operative work when with 80 per cent. of our cases recurring catarrhal inflammations still remain as an important causative factor. In younger subjects with deafness of one or two years' standing, the removal of turbinate pressures, ethmoidal disease or adenoids may be followed by much improvement in the auditory function without special attention to the middle ear, but with those cases giving a history of five, ten or twenty years impairment of hearing we are sure to find that the inflammatory action within the ear and Eustachian tubes will continue if we do not so institute a thorough and persistent course of after treatment.

Chronic catarrhal deafness is a preventable disease. In every one of these patients we will find that there is some habitual transgression of nature's hygienic laws—unseasonable clothing, improper diet, bathing, heating and ventilation, exposure to draughts night or day, too little exercise, and the like. Here is where our greatest effort must be put forth. These errors must be corrected and the bodily surface toughened by cold baths and friction, for when membranes have once been in a state of chronic congestion, dietary and other excesses, or the taking of a slight cold, will produce a profound impression on the weakened blood-vessels.

A few patients afflicted with this disease give no history of nasal catarrh, but we will invariably find the post-nasal and Eustachian membranes in some stage of inflammation, frequently pale and relaxed, but very sensitive. This class need little operative work, but require regular stimulation, as with a light spray of iodol and ether, 2 gr. to the ounce, together with close observation. Their habits must be looked into and so regulated that they are better able to resist colds and throw off congestions.

Assuming that our patients are sensible and intelligent people, it is just and expedient that we go quite into details in explaining nature's method of repair and the different steps of treatment. No further encouragement or promise is necessary if we make the points clear. An ignorant, unreasonable patient is not a favorable one, for they fail to appreciate the obstacles to be overcome and the great need of regular and careful attention.

Surrounded as most patients are by unfavorable climatic influences, and tempted by the good things of life to unhealthful indulgence, we do wrong to encourage them in thinking that they will have no relapses, but we can assure them that these relapses will be much more tractable and easily subdued.

A plan that appears reliable in chronic catarrhal inflammations is to see that each removable cause is taken care of, then after the parts are healed, make a new test of the hearing and begin the second stage of our work. A vapor of camphor and iodine by interrupted jets, applied through the Eustachian catheter serves well a treble purpose of toning up relaxed or atrophied membranes, increasing ossicular mobility and absorbing inflammatory products. These treatments should be gauged according to the individual case in hand,—some every day, some twice a week, but each with the most particular care until sensitiveness of the tubal membranes is removed, the relaxed blood-vessels toned up, and we cease to get more improvement in the hearing.

A rest from active treatment can then be permitted, but the patient should be instructed to report again at the office as soon as an increase in deafness is noted. These periods of rest may become longer and longer until three to six months are allowed.

An interesting feature is that many times after these periods of rest, through nature's good work, we can push the improvement further than at our previous attempt seemed possible, and to a point where the patient is satisfied or perfect hearing established.

We must not expect much improvement in hearing during or soon after the operative stage, but nature's power of regeneration is truly wonderful, if causes for inflammatory action are removed and local tonic application to the membranes are steadily made.

Under the foregoing plan of procedure, many of the cases that have been given an unfavorable prognosis, because they failed to improve with a six or eight weeks' course of treatment, can be taken up and very satisfactory results obtained.

In an article read by the author before the American Rhinological, Laryngological and Otological Society last May, under the title, Modern possibilities in chronic catarrhal deafness, detailed reports of time required and methods used in some obstinate cases, representing three distinct ages and periods in life, were made. Quoting from the same I would again say:

The question does not seem to be so much whether we have an atrophic or hypertrophic condition, but did the deafness primarily occur as a catarrhal inflammation, or is there so much fixation of the ossicles as to preclude a possibility of relief, except through operative procedures?

If examination shows that the trouble be a catarrhal process, and there is a chance of stretching adhesions and absorbing inflammatory products, each year's added experience has given us more courage to make a favorable prognosis.

Many practitioners are opposed to the treatment of deafness in particular, and catarrhal affections in general. This influence is felt in families, and in those cases where prompt, energetic measures are imperative, may become pernicious. Their opposition is honest and comes from the unfavorable prognosis given by authorities for whom they have great respect. We maintain that the conclusions of these authorities were based on experience obtained under auspices much less favorable than the present; their every effort on the ear was hampered by recurring catarrhal inflammations, which today we can in a great measure control.

204 East Jefferson Street.

